# Facilitating action planning within audit and feedback interventions: a mixed-methods process evaluation of an action implementation toolbox in intensive care

**DOI:** 10.1186/s13012-019-0937-8

**Published:** 2019-09-18

**Authors:** Wouter T. Gude, Marie-José Roos-Blom, Sabine N. van der Veer, Dave A. Dongelmans, Evert de Jonge, Niels Peek, Nicolette F. de Keizer

**Affiliations:** 1 Department of Medical Informatics, Amsterdam UMC, Amsterdam Public Health Research Institute, University of Amsterdam, Amsterdam, The Netherlands; 2 Department of Intensive Care Medicine, Amsterdam UMC, University of Amsterdam, Amsterdam, The Netherlands; 3 National Intensive Care Evaluation (NICE) Foundation, Amsterdam, The Netherlands; 4 Centre for Health Informatics, Division of Informatics, Imaging and Data Sciences, Faculty of Biology, Medicine and Health, Manchester Academic Health Science Centre, The University of Manchester, Manchester, UK; 5 Department of Intensive Care Medicine, Leiden University Medical Center, Leiden, The Netherlands; 6 NIHR Greater Manchester Primary Care Patient Safety Translational Research Centre, Manchester Academic Health Science Centre, The University of Manchester, Manchester, UK

**Keywords:** Intensive care, Medical audit, Feedback, Quality improvement, Quality indicators

## Abstract

**Background:**

Audit and feedback (A&F) is more effective if it facilitates action planning, but little is known about how best to do this. We developed an electronic A&F intervention with an action implementation toolbox to improve pain management in intensive care units (ICUs); the toolbox contained suggested actions for improvement. A head-to-head randomised trial demonstrated that the toolbox moderately increased the intervention’s effectiveness when compared with A&F only.

**Objective:**

To understand the mechanisms through which A&F with action implementation toolbox facilitates action planning by ICUs to increase A&F effectiveness.

**Methods:**

We extracted all individual actions from action plans developed by ICUs that received A&F with (*n* = 10) and without (*n* = 11) toolbox for 6 months and classified them using Clinical Performance Feedback Intervention Theory. We held semi-structured interviews with participants during the trial. We compared the number and type of planned and completed actions between study groups and explored barriers and facilitators to effective action planning.

**Results:**

ICUs with toolbox planned more actions directly aimed at improving practice (*p* = 0.037) and targeted a wider range of practice determinants compared to ICUs without toolbox. ICUs with toolbox also completed more actions during the study period, but not significantly (*p* = 0.142). ICUs without toolbox reported more difficulties in identifying what actions they could take. Regardless of the toolbox, all ICUs still experienced barriers relating to the feedback (low controllability, accuracy) and organisational context (competing priorities, resources, cost).

**Conclusions:**

The toolbox helped health professionals to broaden their mindset about actions they could take to change clinical practice. Without the toolbox, professionals tended to focus more on feedback verification and exploring solutions without developing intentions for actual change. All feedback recipients experienced organisational barriers that inhibited eventual completion of actions.

**Trial registration:**

ClinicalTrials.gov, NCT02922101. Registered on 26 September 2016.

**Electronic supplementary material:**

The online version of this article (10.1186/s13012-019-0937-8) contains supplementary material, which is available to authorized users.

Contributions to the literature

- Based on half a century of audit and feedback (A&F) research, we know A&F interventions are more effective if they facilitate action planning, but little is known about how best to do this. In a cluster RCT (published elsewhere), we showed that a toolbox that suggests improvement actions increased A&F effectiveness.
- The current study focused on understanding the mechanism by which the toolbox increased A&F effectiveness. We showed that feedback recipients who used the toolbox progressed through the action planning processes quicker, whilst targeting more areas for improvement.
- We also showed that end-users preferred toolbox actions targeting care processes over those targeting outcomes because the latter require more organisational and cultural changes.
- Our findings contribute to the design of action planning functionalities in A&F interventions and underline which barriers could be overcome by an action implementation toolbox and which barriers would require additional co-interventions.

## Introduction

Audit and feedback (A&F) is an important strategy to close the gap between actual and desired clinical practice, but its observed effects vary greatly across studies [1]. The Cochrane review of 140 randomised controlled trials (RCTs) identified action planning to be an important effect modifier of A&F [1]. However, few A&F trials explicitly described action planning as part of the intervention, and those trials that did appeared to deliver this component of the intervention in various ways [2, 3]. As a result, some recent studies that aimed to reproduce the positive effect of action planning have failed [4]. This indicates that the review’s finding about the positive effect of action planning, derived from indirect comparisons from meta-regressions, is insufficiently reliable and does not inform us how to incorporate action planning as an intervention component to A&F [5, 6]. Conducting head-to-head trials instead, comparing different action planning approaches in addition to providing A&F, would provide useful information to guide the operationalisation of action planning in A&F and other quality improvement interventions [7–9]. Although action planning is a familiar activity in clinical practice, health professionals often lack the time, skills or knowledge to interpret feedback and formulate and plan improvement actions [10]. Providing an action implementation toolbox, containing suggested actions and materials linked to specific potential barriers in the care process, may support this action planning process.

**Fig. 1:**
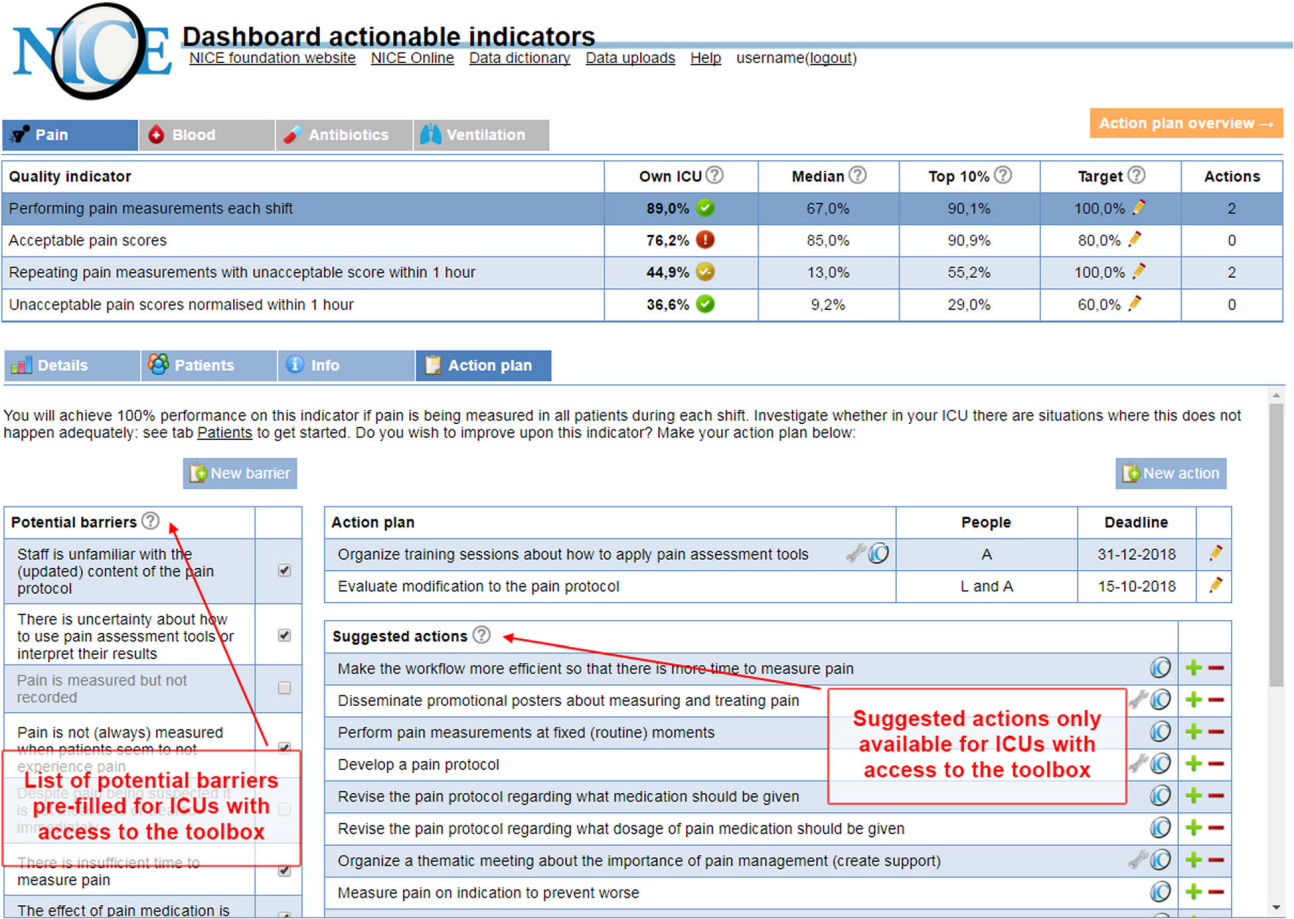
The NICE dashboard displayed an overview of pain management performance (upper part) and four types of pages specific to the selected indicator (lower part). The difference between study groups was only in the action plan page. The *feedback only* group received a blank structured action plan and could record and update potential barriers and intended actions. The action plan for the *feedback with toolbox* group was supplemented with a pre-filled list of potential barriers and suggested actions (indicated by the NICE icon). Some actions included supporting materials (indicated by a wrench icon) available for download. Users could add suggested actions to their action plan and specify their description (plus sign) or hide them if they were not relevant (minus sign)

The Dutch National Intensive Care Evaluation (NICE) foundation developed an electronic A&F intervention to improve pain management practice in Dutch ICUs [11, 12]. In this context, optimal pain management means that pain is measured in every patient in each shift; pain scores are usually acceptable; and if they are not, appropriate treatment is given and pain measurement is repeated within 1 h to evidence that the pain is normalised. The intervention was evaluated in a head-to-head cluster RCT in which all participating ICUs received feedback on four pain management indicators. ICUs were randomised to receive a blank structured action plan with action implementation toolbox (*feedback with toolbox* group) and the other half to receive a blank structured action plan but no toolbox (*feedback only* group) [13]. The toolbox listed potential barriers in the pain management process alongside suggested actions and materials to address these (Fig. 1) [11]. Based on theory and previous empirical research [6, 14–16], we hypothesised that the toolbox would increase the effectiveness of the A&F intervention by helping ICU teams to formulate and implement improvement actions. Consistent with this hypothesis, the trial analysis found absolute improvement on overall pain management performance in both the *feedback with toolbox* group (14.8%; 95% confidence interval [CI], 14.0–15.5) and the *feedback only* group (4.8%; 95% CI, 4.2–5.5), with the *feedback with toolbox* group achieving significantly larger effects than the *feedback only* group (*p* = 0.049) [13].

In this study, we aimed to understand the mechanisms through which the A&F intervention in combination with the action implementation toolbox facilitated ICUs’ action planning processes to inform future A&F and toolbox research. To achieve this, we examined how action planning processes differed between the study arms in terms of the development of action plans, the extent to which those plans were successfully executed and what barriers and facilitators were experienced during these processes.

## Methods

### Study setting and participants

The study was carried out alongside a cluster RCT (ClinicalTrials.gov NCT02922101). Twenty-one ICUs in the Netherlands participated, including 25,141 patient admissions [11, 13]. ICUs were eligible to participate if they (1) had allocated staff to set up a local quality improvement team, consisting of at least one intensivist and one nurse, and—in addition to their regular data upload to the NICE quality registry—(2) had the possibility to submit all data items required for calculating four newly defined pain management indicators [17]. Each ICU identified one team member as the primary contact for the research team during the trial. The medical manager of each ICU signed a consent form to formalise their commitment. ICUs started receiving the intervention between January 2017 and November 2017 for a duration of 6 months. ICUs were aware that there were two variations of the intervention being evaluated, but they were not told what aspect (i.e. the toolbox) was randomised. Figure 1 displays a screenshot of the dashboard and describes how action planning with and without action implementation toolbox took place.

### Theoretical framework

**Fig. 2:**
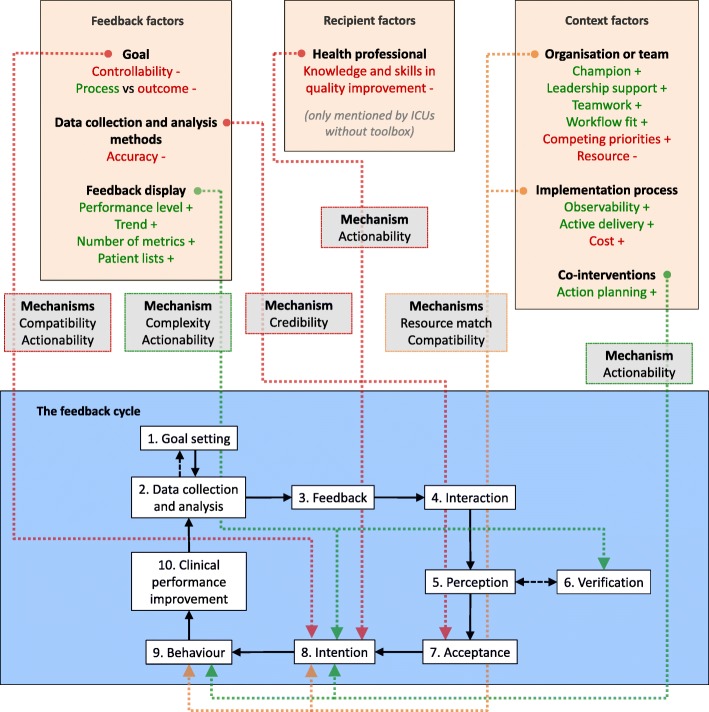
Key findings from the qualitative analysis of the telephone interviews explained by Clinical Performance Feedback Intervention Theory [18]. The identified factors had facilitating (green), inhibiting (red) or mixed (orange) impact on specific processes of the feedback cycle and exerted their effects through various mechanisms. ICU intensive care unit

The recently published Clinical Performance Feedback Intervention Theory (CP-FIT) provides information about how feedback works in practice and what factors may influence its effects [18]. It builds upon 30 pre-existing theories relating to feedback and a meta-synthesis of 65 qualitative evaluation studies of 73 A&F interventions. CP-FIT presents a feedback cycle consisting of a sequence of ten processes: *Goal setting*, *Data collection and analysis*, *Feedback*, recipient *Interaction*, *Perception*, optional *Verification* and *Acceptance* of the feedback, followed by *Intention*, *Behaviour* and *Clinical performance improvement* (see Fig. 2). It proposes that feedback becomes less effective if any of those individual processes fails, causing progress around the cycle to slow or stop (e.g. if feedback recipients do not accept the feedback, they will not develop improvement intentions). Feedback processes’ success or failure is influenced by 70 factors relating to the feedback itself, the recipient of the feedback and the context in which the feedback is delivered. These factors exert their effects via seven explanatory mechanisms of increasing or decreasing the feedback’s *Complexity*, *Relative advantage*, *Resource match*, *Compatibility*, *Credibility*, *Social influence* and *Actionability* [18].

For the current study, we were interested into what extent ICU teams confronted with feedback (*Perception*) react with planning (*Intention*) and completing (*Behaviour*) actions aimed at actually improving practice. Following CP-FIT, ICUs may also plan actions that may not directly lead to *Clinical performance improvement*, such as verifying the feedback’s underlying data (*Verification*, but no *Acceptance*) or exploring possible solutions to the problem (*Acceptance*, but no *Intention*).

### Data collection

We extracted all individual actions from the action plans that ICUs had developed and maintained in the electronic dashboard as part of the intervention. This included the actions’ titles, descriptions, deadlines, assigned people, status (in progress, completed, cancelled), whether they were self-defined or selected from the toolbox and timestamps of any action plan changes (e.g. status update).

We held brief semi-structured telephone interviews with the ICUs’ main contact every 4–6 weeks during the trial to explore which barriers and facilitators ICUs experienced relating to the action planning process. We also verified whether action plans were up-to-date and encouraged ICUs to update the action plan if this was not the case. The structure of the calls was the same for ICUs in both study groups, with the exception that the action implementation toolbox and its contents were not mentioned to ICUs in the *feedback only* group. The interview topic guide is included as Additional file 1. The interviews were conducted by one researcher (WG or MRB) and lasted approximately 10–15 min. Interview notes were emailed to the ICU team for member checking [19].

### Analysis

Two researchers (WG and MRB, who also conducted the interviews) independently coded all actions from the action plans in three steps as described below; discrepancies were resolved through consensus. First, we labelled the individual actions to identify similar actions. We derived labels from the list of toolbox actions as a starting point and iteratively added labels for self-defined actions that did not match any of the pre-defined toolbox actions. Second, we used CP-FIT to identify for each action which feedback cycle process it reflected (i.e. *Verification*, *Acceptance*, *Intention* or *Behaviour*) to indicate its proximity to actual practice change. Third, we grouped those actions reflecting *Intention* or *Behaviour* using the integrated framework for identifying factors that prevent or enable improvements in healthcare professional practice by Flottorp et al. [20] to understand which practice determinants ICUs had targeted for change. Practice determinants are “factors that might prevent or enable improvements in that practice” and have been organised in seven domains: guideline factors (e.g. compatibility of the recommended behaviour), individual health professional factors (e.g. awareness with the recommendation), patient factors (e.g. patient preferences), professional interactions (e.g. communication and influence), incentives and resources (e.g. information system), capacity for organisational change (e.g. leadership) and social, political or legal factors (e.g. payer or funder policies) [20].

We counted the number of actions per feedback cycle process [18] and per targeted practice domain [20] for both study groups. We used chi-square tests to test whether the distribution of actions across the feedback cycle processes and targeted practice domains differed between groups. We also calculated ICUs’ median and interquartile range (IQR) number of planned and completed actions, completion rates and time (in days) taken to complete actions; we tested the difference between groups using the Wilcoxon test. We also counted for each unique action the number of ICUs in both study groups that had planned and/or completed the action during the trial. This allowed us to assess the added value of the toolbox by understanding (1) which actions were most or least often selected, (2) which actions were easy or difficult to complete and (3) to what extent and how toolbox actions differed from self-defined actions.

The interview transcripts were independently coded by two researchers (WG and MRB) using CP-FIT’s codebook [18]. For each barrier or facilitator mentioned by ICUs, we marked which mechanisms they activated and which feedback cycle process of interest (i.e. *Verification*, *Acceptance*, *Intention or Behaviour*) was affected.

## Results

Table 1 displays the baseline characteristics of the 21 ICUs that participated in the trial (10 in the *feedback with toolbox* group and 11 in the *feedback only* group). ICU teams usually consisted of three to five members and included intensivists, nurses and managers. The median baseline pain management performance was, despite randomisation, significantly lower in the *feedback with toolbox* group compared to the *feedback only* group (70.2% vs. 62.5%; *p* = 0.049) [13].

**Table 1:** Characteristics of the 21 ICUs participating in the cluster-randomised controlled trial

ICU characteristic	Feedback with toolbox (*n* = 10)	Feedback only (*n* = 11)
Hospital type, *n* (%)		
Academic	1 (10%)	2 (18%)
Non-academic	9 (90%)	9 (72%)
Number of beds, median (IQR)	12.5 (9.0–15.5)	16.0 (11.0–24.0)
Number of admissions per year, median (IQR)	835 (645–1726)	1048 (757–2046)
Surgical admissions	288 (215–1051)	423 (223–1167)
Medical admissions	533 (313–697)	668 (524–774)
Number of QI team members, median (IQR)	4 (3–5)	4 (3–4)
Baseline pain management performance*, median (IQR)	62.5 (51.6–76.1)	70.2 (53.3–76.0)
Indicator 1—measuring pain each shift	67.8 (61.8–82.3)	81.4 (58.4–87.8)
Indicator 2—acceptable pain scores	87.5 (80.6–91.3)	85.3 (82.7–87.7)
Indicator 3—repeating pain measurements within 1 h	14.4 (5.2–28.7)	13.0 (8.4–19.3)
Indicator 4—normalised pain scores within 1 h	12.3 (4.1–22.6)	9.6 (6.8–15.7)

*ICU* intensive care unit, *IQR* interquartile range, *QI* quality improvement

*The performance composite was measured over the preceding 3 months and defined as the percentage of patient-shift observations during which pain was measured at least once and no unacceptable pain score was observed and/or unacceptable pain scores were followed by a repeated measurement and normalised within 1 h

### Action plans

Action plans contained a total of 234 individual actions (upper part Table 2). Fifty-three (34.6%) actions reflected the *Verification* or *Acceptance* process of the feedback cycle, with the remaining 181 (77.4%) targeting actual practice change. Sixty-five (27.8%) of these were still in progress, reflecting *Intention*, and 116 (49.6%) were completed, reflecting *Behaviour*. The distribution of the actions’ coded positions in the feedback cycle differed significantly between study groups (*p* = 0.012), with 87.3% (128 out of 153) of the actions in the *feedback with toolbox* group reflecting the *Intention* or *Behaviour* process compared to 65.4% (53 out of 81) in the *feedback only* group.

**Table 2:** Comparison of the action plan contents between the *feedback with toolbox* (10 ICUs) and *feedback only* (11 ICUs) group

Action plan contents (all actions)	Feedback with toolbox (*n* = 153)	Feedback only (*n* = 81)	*p* value
Position in the feedback cycle*			0.012
Verification (e.g. check data accuracy, understand the problem)	9 (5.9)	10 (12.3)	
Acceptance (e.g. explore possible solutions)	16 (10.5)	18 (22.2)	
Intention (i.e. active effort to change practice)	49 (32.0)	16 (19.8)	
Behaviour (i.e. practice change)	79 (51.6)	37 (45.7)	
Total number of actions completed	96 (62.7)	51 (63.0)	
Action plan contents (actions reflecting *Intention* or *Behaviour* to change practice)	Feedback with toolbox (*n* = 128)	Feedback only (*n* = 53)	*p* value
Origin			
Selected from the toolbox	104 (81.3)		
Self-defined; similar action available in the toolbox	13 (10.2)	32 (60.4)	
Self-defined; no similar action available in the toolbox	11 (8.6)	21 (39.6)	
Practice domain targeted*			0.331
Guideline	21 (16.4)	6 (11.3)	
Individual health professional	50 (39.1)	29 (54.7)	
Professional interactions	3 (2.3)	1 (1.9)	
Incentives and resources	36 (28.1)	14 (26.4)	
Capacity for organisational change	13 (10.2)	3 (5.7)	
Patient	5 (3.9)	0 (0.0)	
Total number of actions completed	79 (61.7)	37 (69.8)	
Median number of actions planned (IQR)	8 (6.25–14.75)	5 (2.5–5)	0.037
Median number of actions completed (IQR)	4 (2–11)	3 (1–3)	0.142
Median percentage of actions completed (IQR)	59.2 (37.9–94.1)	60 (20–75)	0.479
Median average number of days taken to complete actions (IQR)	56.5 (32.6–125.4)	59.8 (44–95.8)	0.762

Values are *n* (%) of the total number of actions in the study group, except the median (*IQR*; interquartile ranges) values which summarise at the action plan level (equals ICU level)

*Positions in the feedback cycle reflect one of the sequential feedback processes from CP-FIT [18]; practice domains originate from Flottorp et al.’s checklist for identifying practice determinants [20]

Considering only the subset of those actions reflecting *Intention* or *Behaviour* to change practice (lower part Table 2), ICUs in the *feedback with toolbox* group planned a median of 8 (6.25–14.75) actions and completed 4 (IQR, 2–11) actions during the intervention period. They picked 104 (81.3%) of 128 actions from the toolbox whilst the remaining 24 were self-defined. ICUs in the *feedback only* group planned a median of 5 (IQR, 2.5–5) actions and completed 3 (IQR, 1–3); 32 (60.4%) of the 53 actions in the group were equal or similar to actions present in the toolbox. Whereas the median number of planned actions was significantly higher in the *feedback with toolbox* group (*p* = 0.037), this was not the case for the median number of completed actions (*p* = 0.142). In both groups, ICUs completed approximately 60% of their planned actions during the intervention period (*p* = 0.479); each action took them on average 2 months to complete (*p* = 0.762).

In both study groups, actions most often sought practice change in individual health professional factors (39.1% and 54.7%) and resources (28.1% and 26.4%) based on Flottorp et al.’s integrated framework for identifying factors that prevent or enable improvements in healthcare professional practice [20]. The distribution of the actions across targeted practice domains was similar between the two groups (*p* = 0.331) (Table 2). We identified 29 unique actions from the action plans of both study groups (Table 3). Action plans from ICUs in the *feedback with toolbox* group included 26 (89.7%) of these and the *feedback only* group included 19 (65.5%) of these. Ten unique self-defined actions did not match any of the toolbox actions (indicated with an asterisk in Table 3). Two toolbox actions (keep standard prescriptions of pain medication readily available; increase nurses’ autonomy to prescribe pain medication) were not planned by any ICU during the trial.

**Table 3:** Unique actions (*n* = 29) grouped by practice domain with the percentage of ICUs that planned (and completed) these actions

Unique actions grouped by practice domain	% of ICUs that planned (completed) the action	
	*Feedback with toolbox* (*n* = 10)	*Feedback only* (*n* = 11)
Guideline (e.g. clarity of the recommendation, accessibility)	60 (40)	36.4 (27.3)
Revise pain protocol about pain monitoring (e.g. frequency, triggers)	40 (20)	9.1 (0)
Workflow redesign; introduce routine to measuring pain	20 (20)	9.1 (9.1)
Increase protocol accessibility (e.g. electronically, flow chart, pocket card)	20 (10)	9.1 (9.1)
Develop pain protocol	20 (10)	–
Revise pain protocol about pain medication (e.g. drug choice, dosage choice)	10 (10)	27.3 (18.2)
Workflow redesign; check contraindications to pain medications at admission	10 (0)	–
Individual health professional (e.g. knowledge and skills; cognitions)	90 (70)	81.8 (63.6)
Digital newsletter or email	70 (60)	27.3 (27.3)
Educational meeting	70 (50)	9.1 (9.1)
Individual feedback if pain has not been measured	60 (20)	9.1 (9.1)
Promotional poster or message board	40 (30)	36.4 (27.3)
Announcement about pain monitoring during regular staff meeting*	10 (0)	72.7 (63.6)
Quiz about ICU pain management knowledge*	–	9.1 (9.1)
Professional interactions (e.g. communication and influence; team processes)	30 (10)	9.1 (9.1)
Include information on pain status to hand-over moments during shift change	20 (0)	9.1 (9.1)
Collaborate with Acute Pain Service about pain medication after surgery*	10 (10)	–
Incentives and resources (e.g. availability of resources; information system)	100 (80)	63.6 (36.4)
Build or adapt reminder system in the EHR	80 (50)	36.4 (18.2)
Increase completeness of pain measurement data recording in the local EHR	50 (30)	–
Expand order set in EHR (e.g. add pain measurement order at admission)*	40 (40)	18.2 (9.1)
Provide validated pain measurement tools (i.e. NRS, VAS, BPS, CPOT)	30 (20)	–
Adapt EHR form to facilitate pain measurement recording*	10 (10)	18.2 (9.1)
Create performance monitoring tool/dashboard*	10 (0)	9.1 (0)
Nonfinancial incentives (e.g. edible treat) if targets are achieved*	–	9.1 (9.1)
Keep patient-controlled analgesia (PCA) pumps in stock locally at ICU*	–	9.1 (0)
Capacity for organisational change (e.g. mandate, authority, accountability)	40 (40)	27.3 (18.2)
Assign or involve a pain coordinator	40 (40)	18.2 (9.1)
Workflow redesign; increase efficiency to allow more time for measuring pain	10 (10)	–
Expand pain management QI team*	10 (10)	–
Monitoring and feedback on (recording of) pain measurements each shift*	10 (0)	9.1 (9.1)
Monitoring and feedback on appropriateness of prescribed pain medication	10 (0)	–
Patient (e.g. patient needs; preferences)	30 (10)	–
Workflow redesign; monitor/manage factors (e.g. fear) that may worsen pain	20 (0)	–
Workflow redesign; measure pain on patients’ indication	10 (10)	–

*A self-defined action that did not match any toolbox action

### Experienced barriers and facilitators to action planning processes in the feedback cycle

From the interviews, we identified 19 factors (italicised in brackets) that emerged as barriers or facilitators to action planning processes. These factors exerted their effects through five mechanisms (italicised) onto multiple processes in the feedback cycle. Figure 2 displays a schematic overview.

#### Feedback factors

ICUs in both groups felt that the goal of the feedback, adequate pain management, was not always under their control (*Controllability*), e.g. when patients were admitted with unacceptable pain from other departments. Additionally, ICUs found it easier to remind and encourage staff to measure pain more frequently than to make changes to pain medication to prevent or normalise unacceptable pain scores (*Process* vs *outcome*). These factors inhibited ICUs’ improvement intentions by decreasing ICUs’ *Compatibility* with the feedback, and the feedback’s *Actionability*.

The data collection and analysis methods were questioned by some ICUs as they suspected missing pain measurements in the data underlying the feedback, differences in how pain was determined and mismatching shift times (*Accuracy*). This hampered the feedback’s *Credibility* and inhibited ICUs’ acceptance of the feedback.

Low-performance scores on indicators (*Performance level*) in the dashboard facilitated ICUs’ intentions by increasing *Actionability*. The breakdown of performance in a number of relevant subgroups (*Number of metrics*) and patient lists (*Patient lists*) helped ICUs to verify the feedback by reducing *Complexity* and facilitated intention to improve by making it clear how to focus their actions, increasing *Actionability*. For example, subgroup analyses often showed that pain was more prevalent amongst surgical admissions, and pain measurements were often not performed during a patient’s first shift after admission. Also, ICUs reported that the performance charts (*Trend*) reduced the feedback’s *Complexity* by supporting the interpretation of the effects of actions and decisions on whether their action plan needed amending.

#### Recipient factors

Particularly ICUs in the *feedback only* group reported running out of ideas on what actions to take (*Knowledge and skills in quality improvement*), which reduced the *Actionability* and inhibited improvement intentions. Multiple of these ICUs expressed an interest in learning from best practices in high performing ICUs. Conversely, ICUs in the *feedback with toolbox* group mentioned the toolbox gave them good suggestions for improvement.

#### Context factors

With regard to organisational and team factors, many ICUs had one or two people responsible for implementing the intervention locally (*Champion*). They would typically review the feedback in the dashboard, discuss it with the wider team and update their action plan accordingly (*Teamwork*). ICUs mentioned having a manager as an active member of the local QI team (*Leadership support*) as a facilitating factor. Action planning processes were more efficient in ICUs that already had some QI structures in place before the intervention, including a dedicated QI team and structural meetings (*Workflow fit*), which increased *Resource match* and *Compatibility*. In contrast, all ICUs reported experiencing barriers relating to other tasks, events and busy clinical practice (*Competing priorities*), such as the implementation of a new EHR. Limited resources in terms of staff and time (*Resources*) led to actions being delayed or cancelled.

Factors relating to the implementation process exerted their effects through the same mechanisms of *Resource match* and *Compatibility*. ICUs noticed the positive effects of their completed actions (*Observability*), both via the dashboard’s charts and in clinical interactions, which motivated them to continue with their action planning processes. At the same time, observability was hampered by pain measurements being performed but not recorded in the EHR. Recording all pain measurements in the EHR was considered too resource intensive (*Cost*), especially if patients did not seem to be in pain. The telephone interviews (*Active delivery*) stimulated most ICUs to keep reviewing their feedback, reinforce actions that had stagnated, and update their action plans.

The dashboard’s structured action plan facilitated ICUs’ action planning processes (*Action plan*). This was particularly mentioned by ICUs in the *feedback with toolbox* group. Overall, the toolbox was considered to provide useful guidance for improvement: a team member would typically go through the list of suggested actions and pick those that could be beneficial and feasible for their own local practice. A number of suggestions were deemed too generic, simple or inapplicable, but were often still sufficient as a starting point for ICUs to formulate actions more tailored to their local needs.

## Discussion

Our study revealed that ICUs with access to an action implementation toolbox planned more actions to improve practice and targeted a wider range of practice domains than ICUs without toolbox. ICUs without toolbox tended to remain longer in earlier steps of the feedback cycle such as verifications of the feedback data and explorations of possible solutions, rather than targeting actual practice change. However, although ICUs with toolbox also completed more actions during the study period, this difference was not significant. The toolbox facilitated ICUs’ action planning processes by increasing the actionability because it filled a knowledge gap for health professionals; ICUs in the *feedback only* group reported more difficulties in identifying what actions they could take. However, all ICUs still experienced barriers in the feedback (low controllability and accuracy) and context (competing priorities, limited time and staff resources and cost) that were not targeted by the toolbox or other components of the A&F intervention.

ICUs without toolbox typically focused on low effort activities to increasing staff awareness and knowledge using existent structures (e.g. announcements during regular staff meetings) compared to ICUs with toolbox and expressed that they eventually ran out of ideas on what actions to take. This resonates with various studies that have demonstrated that A&F recipients often find it difficult to formulate improvement actions [10, 21–23]. In several previous A&F studies in ICU settings, health professionals have experienced the A&F as insufficiently actionable [24, 25]. In our study, the toolbox increased actionability, leading to more improvement intentions. Furthermore, toolbox ICUs were able to go beyond increasing staff awareness and knowledge to changing existing workflows.

The lack of difference in the number of completed actions may indicate that the toolbox had limited impact on the extent to which improvement intentions were translated into actual practice change (i.e. behaviour). The related organisational barriers that ICUs experienced have been frequently identified in other studies [26]. Since intentions to change practice are meaningless if they cannot be translated into action, more efforts are needed to facilitate health professionals in this translation. Most of ICUs’ actions targeted care processes (i.e. increasing the frequency of pain measurements) rather than outcomes (i.e. reducing pain). It is a common finding that processes are more commonly targeted than outcomes [27], which often relates to their actionability and controllability, yet those processes may have only an indirect link to improving patient outcomes. Some of the toolbox actions to specifically improve outcomes were hardly or never selected by ICUs, such as increasing nurses’ autonomy to prescribe pain medication. In this setting, such actions may not have been selected due to their invasiveness, consequences for the organisational context and inertia to change traditional divisions of labour (that is, pain medication is prescribed by intensivists, whilst nurses are responsible for monitoring pain and administering the drugs) [28, 29].

### Implications for practice and research

Our findings imply that providing recipients of A&F interventions with practical suggestions for improvement actions is a promising approach to increasing the effectiveness of A&F by making it more actionable. Feedback actionability is known to substantially contribute to the success of interventions [30, 31], but has proven difficult to operationalise in practice [4, 6, 24, 25]. We found that presenting suggested actions for improvement in a toolbox integrated within an electronic, structured action plan is a feasible and potentially less resource-intensive alternative to, for example, educational outreach visits [32]. Some of the remaining barriers to the action planning we identified (e.g. controllability) could be overcome by improving the toolbox contents, whereas others (particularly those relating to the organisational context) may require other co-interventions.

ICUs selected a wide variety of toolbox actions and added a range of self-defined actions that did not match any of the pre-defined toolbox actions. This confirms that different A&F recipients may have different quality improvement needs [33]. We recommend that in the future A&F interventions with action toolbox should, similar to the toolbox in this study, enable recipients to further tailor the toolbox to local context by adding self-defined actions and hide toolbox actions or amend their description in order to tailor the toolbox to their local context.

We derived the toolbox content from guidelines, literature and expert opinion [11, 12]. Although participants considered this useful, they expressed an additional interest in best practices from high performers. Future A&F studies could therefore consider adding “best practice-based” actions to complement toolbox actions derived from other sources and dynamically update the toolbox by further refining actions with low completion rates.

### Strengths and limitations

Our study has several strengths. Whereas the head-to-head RCT had previously demonstrated *that* the toolbox was effective in improving ICU pain management [13], the current study helped us understand the underlying mechanisms for *how* it was effective. This contributes to expanding our knowledge about how action planning in A&F interventions can be facilitated [7]. The electronic nature of our A&F intervention enabled us to study mechanisms in detail and unobtrusively, whilst including all participants [34, 35]. Usage data that are a by-product of using digital A&F interventions in real-world settings is largely underutilised; they also informed the telephone interviews. This created a synergistic mixed-methods approach in which quantitative and qualitative complemented as well as strengthened each other [36].

Some limitations warrant discussion. The toolbox made it easier for ICUs to select (many) actions by one click of the mouse, whilst ICUs without toolbox had to formulate actions themselves. In addition, self-defined actions often differed in “load” and potential impact. This hampered a straightforward comparison on the number of actions between groups. We addressed this by harmonising individual actions based on their content during the first step of our coding procedure. Furthermore, telephone interviews often prompted ICUs to update their plans. As a result, the recorded number of days needed to complete actions is likely overestimated. Next, we did not assess which suggested actions from the toolbox were already implemented in practice before the start of the intervention, which limited our interpretation of why particular actions were not selected by ICUs. Finally, the contents of the toolbox were developed specifically for Dutch ICU setting, and the feasibility of the toolbox’ suggested actions may not be generalisable to other settings.

## Conclusion

An action implementation toolbox containing suggested actions for practice change increases the actionability of A&F interventions, prompting recipients to initiate more changes and across a wider range of practice determinants. The toolbox helped health professionals to broaden their mindset about actions they could take to improve practice. Without the toolbox, professionals tended to focus more on feedback verification and exploring solutions without developing intentions for actual change. All feedback recipients experienced barriers that inhibited completion of actions. Whereas some of those barriers may be overcome when appropriately addressed by a toolbox, other barriers, often relating to the organisational context, will require co-interventions.

## Additional file

Additional file 1: Standards for Reporting Implementation Studies: the StaRI checklist for completion. (DOCX 78 kb)

## Data Availability

The datasets used and/or analysed during the current study are available from the corresponding author on reasonable request.

## Abbreviations

A&F: Audit and feedback
cRCT: Cluster-randomised controlled trial
ICU: Intensive care unit
IQR: Interquartile range
NICE: National Intensive Care Evaluation foundation
SD: Standard deviation
